# Supplementary material for: Vitamin D status modulates mitochondrial oxidative capacities in skeletal muscle: role in sarcopenia
Source: Commun Biol. 2022 Nov 24;5:1288. doi: 10.1038/s42003-022-04246-3 (PMC9700804; doi:10.1038/s42003-022-04246-3)
Supplement: Supplementary file 2 — Supplementary figures and tables [file 42003_2022_4246_MOESM2_ESM.pdf]

Supplementary Figures and tables

Vitamin D status modulates mitochondrial oxidative capacities in skeletal muscle: role in sarcopenia.

Jérôme Salles[1], Audrey Chanet[1], Christelle Guillet[1], Anouk MM. Vaes[2], Elske M. Brouwer-Brolsma[2], Christophe Rocher[3], Christophe Giraudet[1], Véronique Patrac[1], Emmanuelle Meugnier[4], Christophe Montaurier[1], Philippe Denis[1], Olivier Le Bacquer[1], Adeline Blot[5], Marion Jourdan[6], Yvette Luiking[6], Matthew Furber[6], Miriam Van Dijk[6], Nicolas Tardif[7], Yves Boirie Y[1,8], Stéphane Walrand[1,8]*.

[1]Université Clermont Auvergne, INRA, UNH, CRNH Auvergne, 63000 Clermont-Ferrand, France

[2]Wageningen University, Human Nutrition, Wageningen, The Netherlands

[3]Laboratoire de Biogenèse Membranaire - UMR 5200 CNRS, Université de Bordeaux, 33140 Villenave d'Ornon, France.

[4]Univ Lyon, CarMeN Laboratory, INSERM, INRAE, INSA Lyon, Université Claude Bernard Lyon 1, 69310 Pierre-Bénite, France.

[5]CHU Clermont-Ferrand, Centre de Recherche en Nutrition Humaine Auvergne, 63000 Clermont-Ferrand, France.

[6]Specialized Nutrition, Danone Nutricia Research, P.O. Box 80141, 3584 CT Utrecht, The Netherlands.

[7]Division of Perioperative Medicine and Intensive Care, Karolinska University Hospital, Huddinge, Sweden.

[8]CHU Clermont-Ferrand, Service Nutrition Clinique, 63000 Clermont-Ferrand, France.

Supplementary Table 1

Vitamin D-dependent parameters in control (n=7) and vitamin D-depleted (n=9) old rats.

	**Control**	**Vitamin D-depleted**
Calcium (mg/l)	101.6±1.0	103.5±2.1
Phosphorus (mg/l)	52.05±7.80	43.54±3.44
PTH (pg/ml)	376.4±51.1	347.8±27.7

Supplementary Table 2

Tissue weights in control (n=7) and vitamin D-depleted (n=9) old rats.

	**Control**	**Vitamin D-depleted**
Quadriceps (g)	3.21±0.23	2.84±0.21
Gastrocnemius (g)	1.78±0.14	1.56±0.09
Heart (g)	1.89±0.09	1.71±0.05
Liver (g)	13.6±0.8	13.8±0.1.1

Supplementary Table 3

Serum 25(OH)D concentrations and body composition in control (n=10) and vitamin D-depleted (n=9) mice at 24 months of age (Mouse experiment 1).

	**Control**	**Vitamin D-depleted**
25(OH)D (nM)	57.4 ± 5.3	< 0.04
Weight (g)	42.1 ± 1.6	43.0 ± 2.1
Fat (%)	32.0 ± 1.3	31.3 ± 1.8
Lean mass (g)	28.0 ± 1.1	29.3 ± 1.0

Serum 25(OH)D concentrations were below the lower limit of quantitation (LLQ < 4 nmol/L) in vitamin D-depleted mice.

Supplementary Table 4

List of significant differentially expressed genes in muscle plantaris from vitamin D-depleted old rats *versus* control old rats associated with GO term 'mitochondrion' (GO:0005739)

**Probe**	**Gene**	**Accession Number**	**Description**	**Fold change**
A_64_P044470	***Mrpl50***	NM_001108665	mitochondrial ribosomal protein L50 (Mrpl50), mRNA	**0.903**
A_64_P131427	***Tomm6***	ENSRNOT00000070805	Tomm6 protein	**0.900**
A_64_P033367	***Mff***	NM_001271284	mitochondrial fission factor (Mff), transcript variant 1, mRNA	**0.898**
A_44_P229568	***Zadh2***	NM_001106129	zinc binding alcohol dehydrogenase, domain containing 2 (Zadh2), mRNA	**0.874**
A_64_P097527	***Pdhx***	FQ232219	gb|Rattus norvegicus TL0AEA66YJ18 mRNA sequence.	**0.867**
A_44_P340316	***Ndufaf7***	NM_001008318	NADH dehydrogenase (ubiquinone) complex I, assembly factor 7 (Ndufaf7), mRNA	**0.864**
A_44_P409729	***Opa1***	NM_133585	optic atrophy 1 (Opa1), mRNA	**0.861**
A_64_P014427	***Pla2g15***	NM_001004277	phospholipase A2, group XV (Pla2g15), mRNA	**0.859**
A_64_P150696	***Araf***	NM_022532	v-raf murine sarcoma 3611 viral oncogene homolog (Araf), transcript variant 1, mRNA	**0.859**
A_44_P197086	***Rpusd4***	NM_001025284	RNA pseudouridylate synthase domain containing 4 (Rpusd4), mRNA	**0.859**
A_44_P925867	***Napg***	NM_001107384	N-ethylmaleimide-sensitive factor attachment protein, gamma (Napg), mRNA	**0.854**
A_44_P527134	***Oxr1***	NM_001197907	oxidation resistance 1 (Oxr1), transcript variant 1, mRNA]	**0.851**
A_64_P164449	***Ndufa10l1***	NM_182671	NADH dehydrogenase (ubiquinone) 1 alpha subcomplex 10-like 1 (Ndufa10l1), mRNA	**0.848**
A_43_P18208	***Pitrm1***	NM_001107363	pitrilysin metallopeptidase 1 (Pitrm1), mRNA	**0.847**
A_64_P015502	***Ide***	NM_013159	insulin degrading enzyme (Ide), mRNA	**0.842**
A_44_P370281	***Herc2***	NM_001107520	HECT and RLD domain containing E3 ubiquitin protein ligase 2 (Herc2), mRNA	**0.842**
A_44_P219668	***Acat1***	NM_017075	acetyl-CoA acetyltransferase 1 (Acat1), mRNA	**0.840**
A_44_P555418	***Gcsh***	NM_133598	glycine cleavage system protein H (aminomethyl carrier) (Gcsh), mRNA	**0.839**
A_44_P415901	***Mrpl15***	NM_001106633	mitochondrial ribosomal protein L15 (Mrpl15), mRNA [NM_001106633]	**0.838**
A_42_P562202	***Atpaf1***	NM_001107959	ATP synthase mitochondrial F1 complex assembly factor 1 (Atpaf1), mRNA	**0.838**
A_44_P149406	***Cbr4***	NM_182672	carbonyl reductase 4 (Cbr4), mRNA	**0.837**
A_64_P075938	***Cox11***	NM_001109575	cytochrome c oxidase assembly homolog 11 (yeast) (Cox11), mRNA	**0.831**
A_44_P130641	***Txn2***	NM_053331	thioredoxin 2 (Txn2), mRNA [NM_053331]	**0.828**
A_64_P128164	***Mrpl47***	NM_001037183	mitochondrial ribosomal protein L47 (Mrpl47), mRNA	**0.828**
A_64_P071483	***Cox7a2***	NM_022503	cytochrome c oxidase subunit VIIa polypeptide 2 (Cox7a2), mRNA	**0.819**
A_42_P782490	***Zfhx3***	ENSRNOT00000019408	Protein Zfhx3	**0.816**
A_43_P17088	***Agpat5***	NM_001134744	1-acylglycerol-3-phosphate O-acyltransferase 5 (Agpat5), mRNA	**0.808**
A_64_P143307	***Rfk***	NM_001014106	riboflavin kinase (Rfk), mRNA	**0.807**
A_44_P480222	***Mapk9***	NM_017322	mitogen-activated protein kinase 9 (Mapk9), transcript variant 1, mRNA	**0.806**
A_64_P006320	***RGD1303003***	NM_001004225	homolog of zebrafish ES1 (RGD1303003), mRNA	**0.804**
A_44_P210202	***Polg***	NM_053528	polymerase (DNA directed), gamma (Polg), mRNA	**0.798**
A_64_P045716	***Hsp90ab1***	NM_001004082	heat shock protein 90 alpha (cytosolic), class B member 1 (Hsp90ab1), mRNA	**0.789**
A_64_P039016	***XM_224716***	XM_224716	gb|Rattus norvegicus similar to GTP binding protein 3 (LOC290633), mRNA	**0,789**

A_44_P413701	***Mdh2***	NM_031151	malate dehydrogenase 2, NAD (mitochondrial) (Mdh2), mRNA	**0,789**
A_42_P472375	***Pdk2***	NM_030872	pyruvate dehydrogenase kinase, isozyme 2 (Pdk2), mRNA	**0,785**
A_43_P11430	***Adsl***	NM_001130503	adenylosuccinate lyase (Adsl), mRNA	**0,778**
A_64_P013491	***Idh3a***	NM_053638	isocitrate dehydrogenase 3 (NAD+) alpha (Idh3a), mRNA	**0,777**
A_42_P627330	***Mrps35***	NM_001106628	mitochondrial ribosomal protein S35 (Mrps35), mRNA [NM_001106628]	**0,773**
A_44_P398148	***Sh3bp5***	NM_054011	SH3-domain binding protein 5 (BTK-associated) (Sh3bp5), mRNA	**0,757**
A_64_P016398	***ENSRNOT00000050750***	ENSRNOT00000050750	L-lactate dehydrogenase	**0,751**
A_44_P1002280	***RGD1302996***	NM_213610	hypothetical protein MGC:15854 (RGD1302996), mRNA	**0,747**
e	***Comt***	NM_012531	catechol-O-methyltransferase (Comt), mRNA	**0,746**
A_42_P763553	***Opa3***	NM_001107486	optic atrophy 3 (Opa3), mRNA	**0,745**
A_44_P552915	***Cpt1c***	NM_001034925	carnitine palmitoyltransferase 1c (Cpt1c), mRNA	**0,745**
A_64_P097603	***Mtus1***	NM_178093	microtubule associated tumor suppressor 1 (Mtus1), mRNA	**0,742**
A_64_P045339	***Kcnj11***	NM_031358	potassium inwardly rectifying channel, subfamily J, member 11 (Kcnj11), mRNA	**0,725**
A_64_P025351	***ENSRNOT00000015332***	ENSRNOT00000015332	Pyruvate kinase isozymes M1/M2	**0,721**
A_64_P145878	***Casq1***	NM_001159594	calsequestrin 1 (fast-twitch, skeletal muscle) (Casq1), mRNA	**0,701**
A_44_P231269	***Pdk4***	NM_053551	pyruvate dehydrogenase kinase, isozyme 4 (Pdk4), mRNA	**0,689**
A_44_P175864	***Trit1***	NM_001108676	tRNA isopentenyltransferase 1 (Trit1), mRNA	**0,670**
A_64_P024077	***Pkm***	NM_053297	pyruvate kinase, muscle (Pkm), mRNA	**0,544**

Supplementary Table 5

Primer sequences for the quantitative analysis of gene expression and mtDNA content in rat and mice tissues, C2C12 myotubes and human primary myotubes.

	Gene name	Forward and reverse primers
Rat	*ND1* (Mitochondrial DNA)<br>Mitochondrially encoded NADH:Ubiquinone oxidoreductase core subunit 1	*For 5'*-TGGTGTACTCGGCTATGAAGAA-*3'*<br>*Rev 5'*-CAGGCTTTAACGTCGAATACG-*3'*
	Beta-actin (Genomic DNA)	*For 5'*-GGGATGTTGCTCCAACCAA-*3'*<br>*Rev 5'*-GCGCTTTTGACTCAAGGATTTAA-*3'*
	COX IV<br>Cytochrome c oxidase subunit IV	*For 5'*-TGGGAGTGTTGTGAAGAGTGA-*3'*<br>*Rev 5'*-GCAGTGAAGCCGATGAAGAAC-*3'*
	CPT1b<br>Carnitine palmitoyltransferase-1b	*For 5'*-CGTGCTTCTGCCCACTCTA-*3'*<br>*Rev 5'*-TCATGTATCGCCGCAAACT-*3'*
	FIS1<br>Mitochondrial fission 1 protein	*For 5'*-GCAACTACCGGCTCAAGG-*3'*<br>*Rev 5'*-CATGGCCTTGTCAATCAGG-*3'*
	MFN1<br>Mitofusin-1	*For 5'*-GGCTGTCAGAGCCTATCTTTCA-*3'*<br>*Rev 5'*-GATGCCAATCCTGTGATCAGT-*3'*
	MFN2<br>Mitofusin-2	*For 5'*-TCCTGGGCCCTAAGAATAGC-*3'*<br>*Rev 5'*-GAGAGGACGCTGAACCTGAT-*3'*
	NRF1<br>Nuclear respiratory factor 1	*For 5'*-TTATTCTGCTGTGGCTGATGG-*3'*<br>*Rev 5'*-CCTCTGATGCTTGCGTCGTCT-*3'*
	NRF2<br>Nuclear respiratory factor 2	*For 5'*-CACCACACTCAACATTTCGG-*3'*<br>*Rev 5'*-TGGAGATGCAATTGCTCAGT-*3'*
	PPARα<br>Peroxisome proliferator-activated receptor alpha	*For 5'*-TGGAGTCCACGCATGTGAAG-*3'*<br>*Rev 5'*-CGCCAGCTTTAGCCGAATAG-*3'*
	PPARβ<br>Peroxisome proliferator-activated receptor beta	*For 5'*-CATTGCCGCCATCATTCTGT-*3'*<br>*Rev 5'*-CTGCATCATCTGCGCGTGTT-*3'*
	PGC1α<br>Peroxisome proliferator-activated receptor gamma coactivator 1-alpha	*For 5'*-AGTTTTTGGTGAAATTGAGGAAT-*3'*<br>*Rev 5'*-TCATACTTGCTCTTGGTGGAAGC-*3'*
	PGC1β<br>Peroxisome proliferator-activated receptor gamma coactivator 1-beta	*For 5'*-ACTATGATCCCACGTCTGAAGAGTC-*3'*<br>*Rev 5'*-CCTTGTCTGAGGTATTGAGGTATTC-*3'*
	TFAM<br>Mitochondrial transcription factor A	*For 5'*-CTGCTTTTCATCATGAGACAG-*3'*<br>*Rev 5'*-GAAAGCACAAATCAAGAGGAG-*3'*

	UCP3<br>Uncoupling protein 3	*For 5'*-GAACTGGAGGCGAGAGGAAA-*3'*<br>*Rev 5'*-GTCATAGGTCACCATCTCAG-*3'*
	RPLP0<br>Ribosomal protein, large, P0	*For 5'*-GTGTTTGACAATGGCAGAT-*3'*<br>*Rev 5'*-ACAGACGCTGGCCACATT-*3'*
**C2C12 myotubes and mice**	*ND1* (Mitochondrial DNA)<br>Mitochondrially encoded NADH:Ubiquinone oxidoreductase core subunit 1	*For 5'*-GGCCCCCTTCGACCTGACAGA-*3'*<br>*Rev 5'*-TAACGCGAATGGGCCGGCTG-*3'*
	Beta-actin (Genomic DNA)	*For 5'*-TACAGCTTCACCACCACAGC-*3'*<br>*Rev 5'*-AAGGAAGGCTGGAAAAGAGC-*3'*
	COX IV<br>Cytochrome c oxidase subunit IV	*For 5'*-TGGGAGGTTGTGAAGAGTGA-*3'*<br>*Rev 5'*-GCAGTGAAGCCGATGAAGAAC-*3'*
	CPT1b<br>Carnitine palmitoyltransferase-1b	*For 5'*-TGGGACTGGTCGATTGCATC-*3'*<br>*Rev 5'*-TCAGGGTTTGTCGGAAGAAGAA-*3'*
	NDUFB5<br>NADH:Ubiquinone oxidoreductase subunit B5	*For 5'*-CCTGGCTATCCTCCAGATTG-*3'*<br>*Rev 5'*-CGCATCAGCCTTCGAACT-*3'*
	NRF1<br>Nuclear respiratory factor 1	*For 5'*-GCTGCTGCTGTGGCAACAGG-*3'*<br>*Rev 5'*-TTGGGTTTGGAGGGTGAGAT-*3'*
	NRF2<br>Nuclear respiratory factor 2	*For 5'*-GGGGAACAGAACAGGAAACA-*3'*<br>*Rev 5'*-CCGTAATGCACGGCTAAGTT-*3'*
	PGC1α<br>Peroxisome proliferator-activated receptor gamma coactivator 1-alpha	*For 5'*-GAAGTGGTGTAGCGACCAATC-*3'*<br>*Rev 5'*-AATGAGGGCAATCCGTCTTCA-*3'*
	PGC1β<br>Peroxisome proliferator-activated receptor gamma coactivator 1-beta	*For 5'*-ACTATGATCCCACGTCTGAAGAGTC-*3'*<br>*Rev 5'*-CCTTGTCTGAGGTATTGAGGTATTC-*3'*
	TFAM<br>Mitochondrial transcription factor A	*For 5'*-CCAAAAAGACCTCGTTCAGC-*3'*<br>*Rev 5'*-ATGTCTCCGGATCGTTTCAC-*3'*
	UCP3<br>Uncoupling protein 3	*For 5'*-GCTAGACGCACAGCTTCCTC-*3'*<br>*Rev 5'*-GTTCCTTTGCTGCCTATGGA-*3'*
	VDR<br>Vitamin D receptor	*For 5'*-TCGCCCCTGCTCCTTCAG -*3'*<br>*Rev 5'*-TTGGTGATGCGGCAATCTC-*3'*
	HPRT<br>Hypoxanthine phosphoribosyltransferase	*For 5'*-AGTTGAGAGATCATCTCCAC-*3'*<br>*Rev 5'*-TTGCTGACCTGCTGGATTAC-*3'*
	RPLP0<br>Ribosomal Protein, Large, P0	*For 5'*-GTGTTTGACAATGGCAGAT-*3'*<br>*Rev 5'*-ACAGACGCTGGCCACATT-*3'*
**Human primary myotubes**	*COX IV*<br>Cytochrome c oxidase subunit IV	*For 5'*-TGGGAGGTTGTGAAGAGTGA-*3'*<br>*Rev 5'*-GCAGTGAAGCCGATGAAGAAC-*3'*

CPT1b	*For 5'*-TACAACAGGTGGTTTGACA-*3'*
Carnitine palmitoyltransferase-1b	*Rev 5'*-CAGAGGTGACCAATGATG-*3'*
NRF1	*For 5'*-GCTGCTGCTGTGGCAACAGG-*3'*
Nuclear respiratory factor 1	*Rev 5'*-TTGGGTTTGGAGGGTGAGAT-*3'*
NRF2	*For 5'*-GGGGAACAGAACAGGAAACA-*3'*
Nuclear respiratory factor 2	*Rev 5'*-CCGTAATGCACGGCTAAGTT-*3'*
PGC1α	*For 5'*-GAAGTGGTGTAGCGACCAATC-*3'*
Peroxisome proliferator-activated receptor gamma coactivator 1-alpha	*Rev 5'*-AATGAGGGCAATCCGTCTTCA-*3'*
PGC1β	*For 5'*-ACTATGATCCCACGTCTGAAGAGTC-*3'*
Peroxisome proliferator-activated receptor gamma coactivator 1-beta	*Rev 5'*-CCTTGTCTGAGGTATTGAGGTATTC-*3'*
TFAM	*For 5'*-CCAAAAAGACCTCGTTCAGC-*3'*
Mitochondrial transcription factor A	*Rev 5'*-ATGTCTCCGGATCGTTTCAC-*3'*
RPLP0	*For 5'*-GTGTTTGACAATGGCAGAT-*3'*
Ribosomal protein, large, P0	*Rev 5'*-ACAGACGCTGGCCACATT-*3*

Supplementary Figure 1: Vitamin D deficiency did not change energy intake and spontaneous activity in old rats.

(a) 24-hour energy intake, (b) energy intake during the light period, and (c) energy intake during the dark period in vitamin D-depleted old rats (n=5) and control old rats (n=5). (d) 24-hour spontaneous activity, (e) spontaneous activity during the light period, and (f) spontaneous activity during the dark period in vitamin D-depleted old rats (n=5) and control old rats (n=5). Data are expressed as means ± SEM. Differences between groups were analyzed using an unpaired t-test.

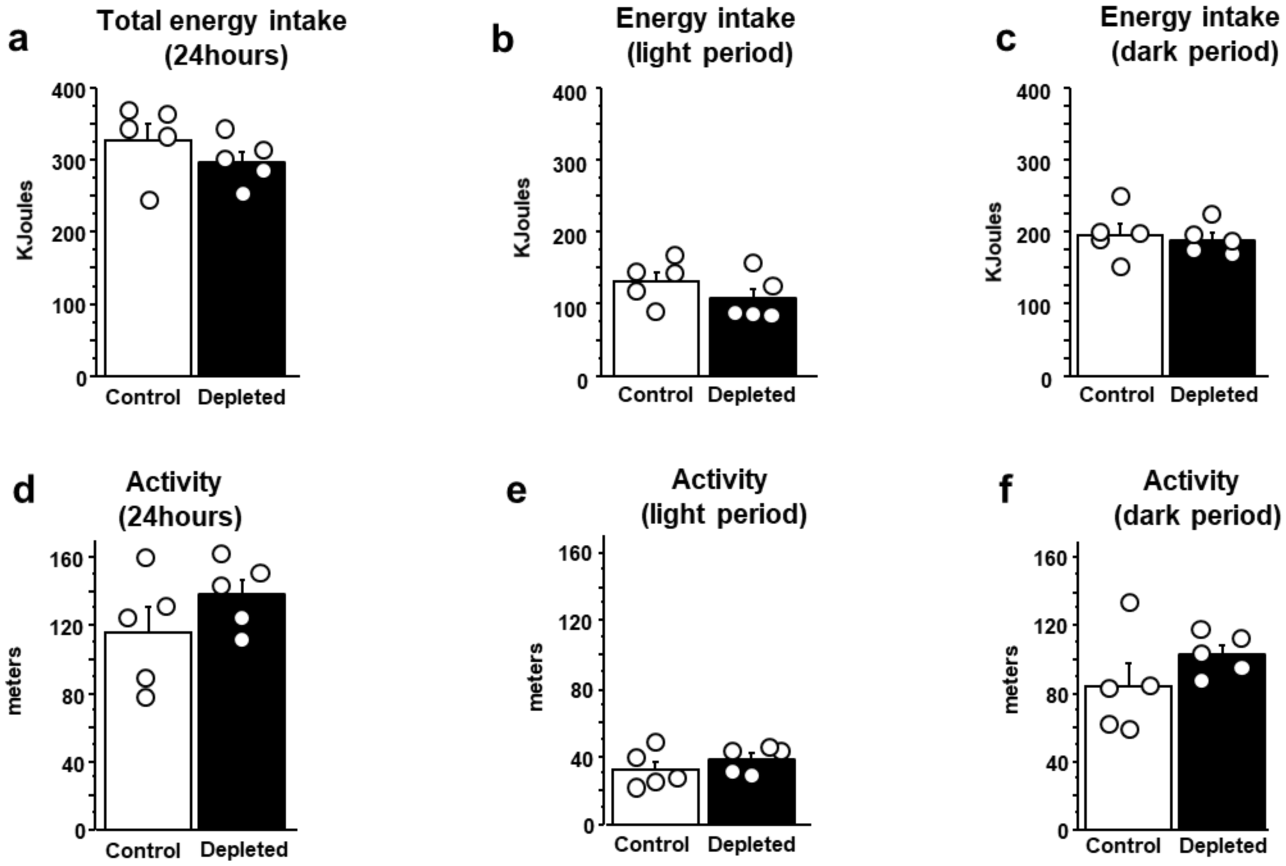

Supplementary Figure 2: Vitamin D regulates expression levels of genes involved in several functions in rat muscles and C2C12 skeletal muscle cells.

DAVID was used for Gene Ontology (GO) enrichment analysis of significant differentially expressed genes (DEGs) in plantaris muscles from old rats ($p<0.05$) and in C2C12 myotubes (adjusted p-values <0.05) according to the vitamin D status. The top 10 significant enriched GO terms of the DEGs upregulated in response to vitamin D deficiency in rat plantaris muscle (a) and the top 10 significant enriched gene ontology terms of the DEGs downregulated in response to vitamin D supplementation in C2C12 myotubes (b) are presented.

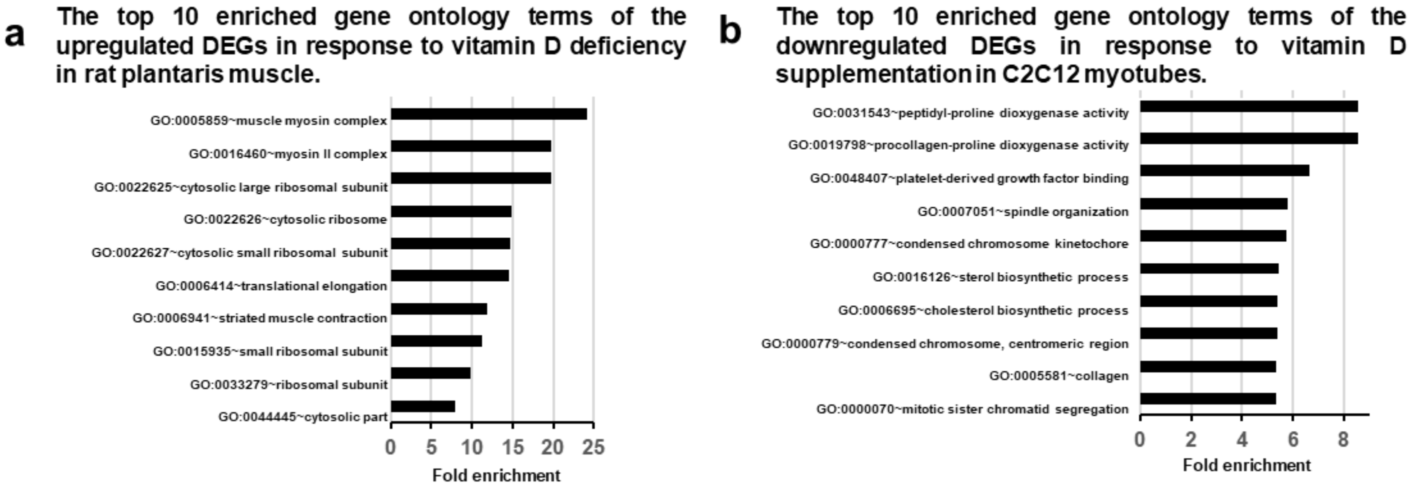

Supplementary Figure 3: Tamoxifen administration does not affect liver, kidney and intestine VDR gene expression in *HSA-MCM-VDR*fl/fl transgenic mice.

Vitamin D receptor (*VDR*) transcript expression levels in (a) liver, (b) kidney and (c) small intestine from tamoxifen-treated *HSA-MCM-VDR*fl/fl mice (n=4-5) and corn oil vehicle-treated *HSA-MCM-VDR*fl/fl mice (n=3-5) (Mouse experiment 2). mRNA levels were normalized to *GAPDH*, *RPLP0* and *β-actin* in liver, kidney and small intestine, respectively. Data are expressed as means ± SEM and as fold change vs. corn oil-treated group value.

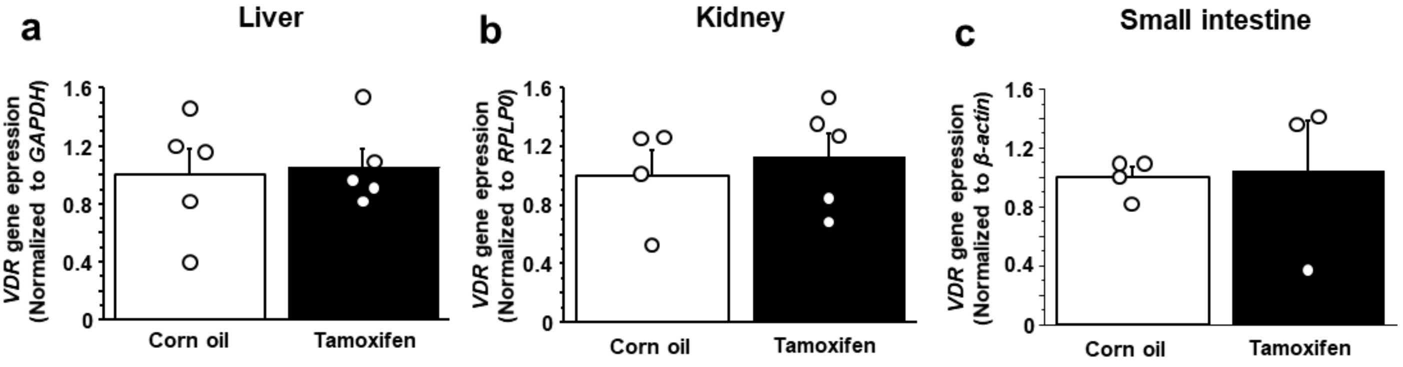

Supplementary Figure 4: Uncropped and unedited blot/gel images.

Figure 3c

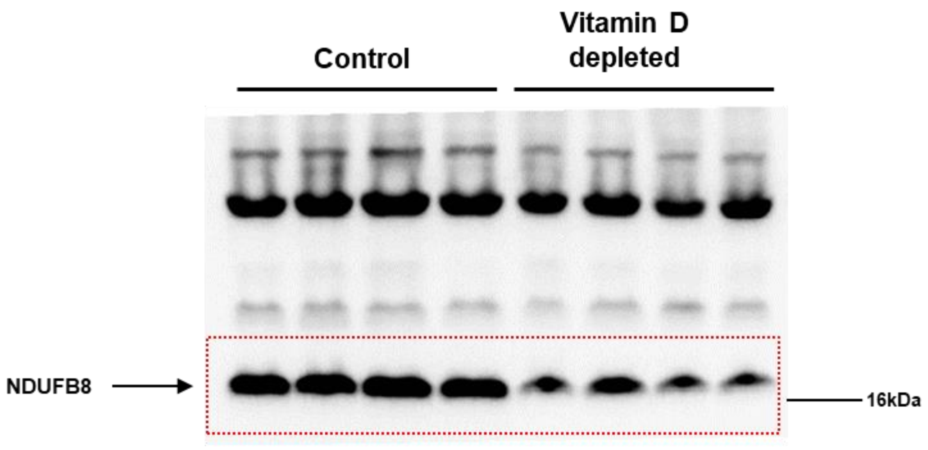

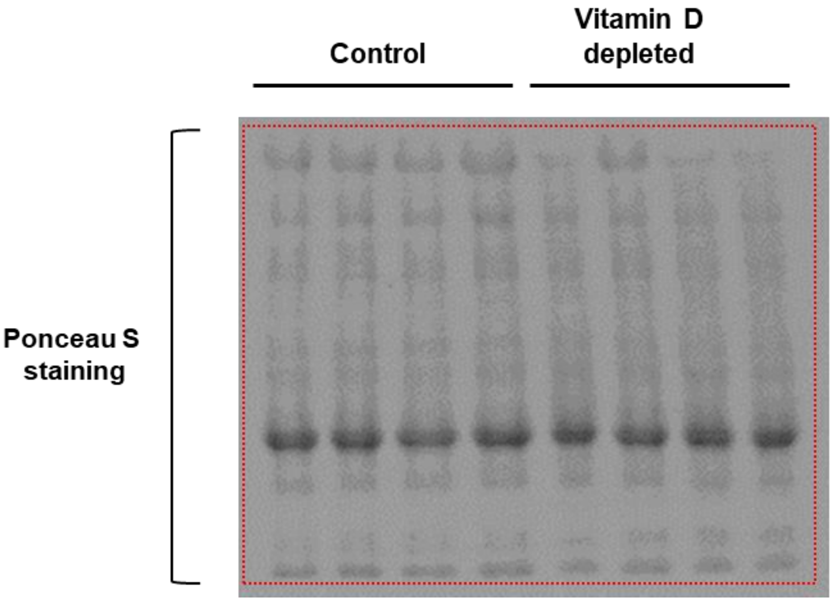

Figure 3e

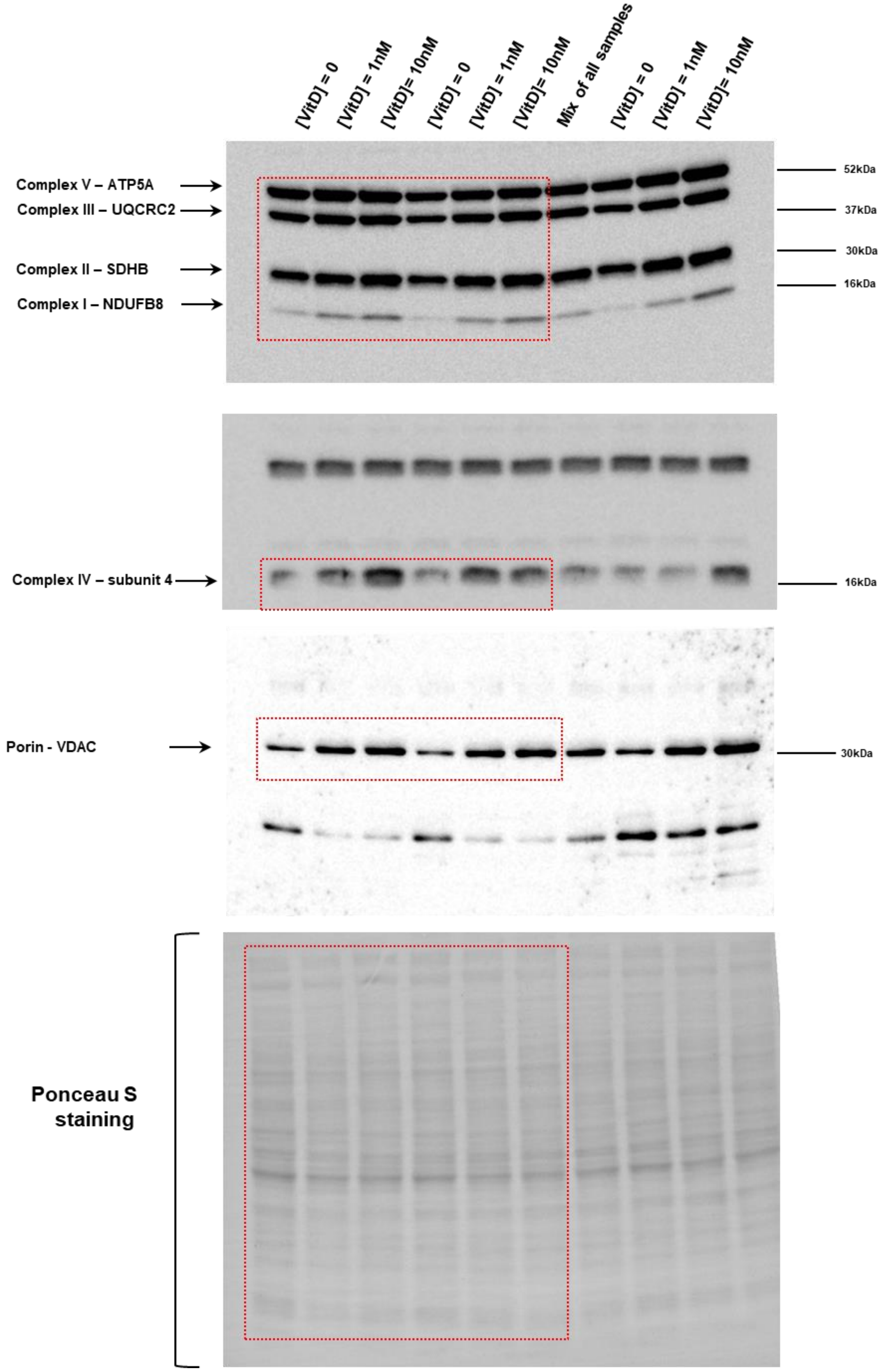
[VitD] = 0
[VitD] = 1nM
[VitD]= 10nM
[VitD] = 0
[VitD] = 1nM
[VitD]= 10nM
Mix of all samples
[VitD] = 0
[VitD] = 1nM
[VitD]= 10nM
Complex V – ATP5A
Complex III – UQCRC2
Complex II – SDHB
Complex I – NDUFB8
52kDa
37kDa
30kDa
16kDa
Complex IV – subunit 4
16kDa
Porin - VDAC
30kDa
Ponceau S staining

Figure 5h

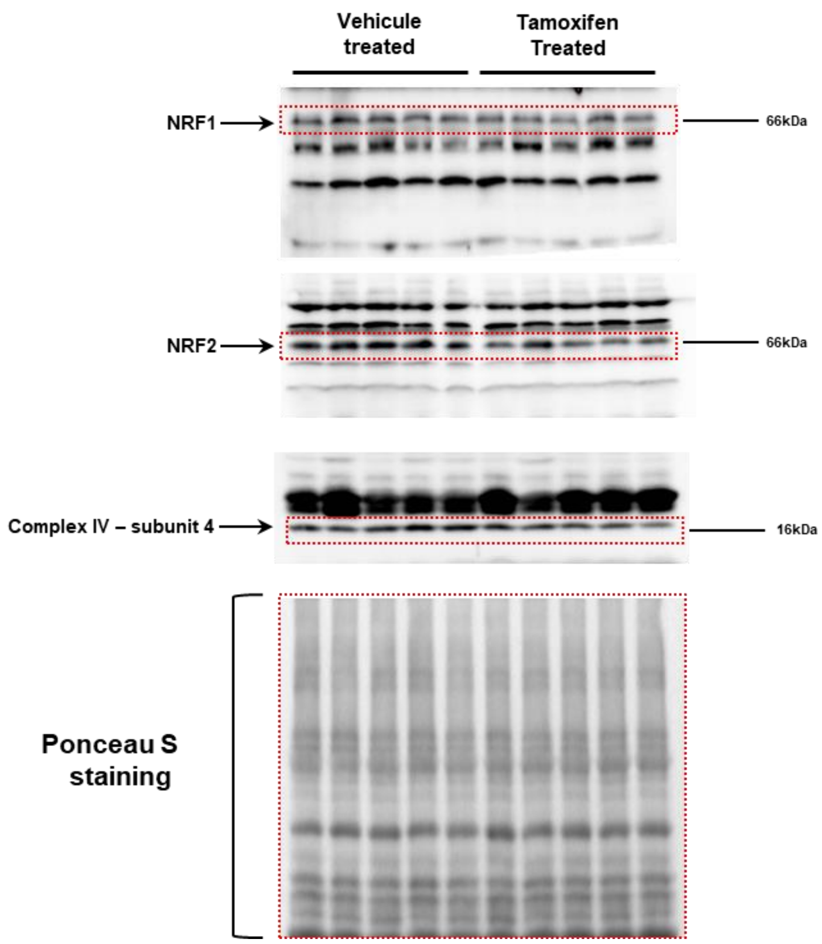
Vehicule treated
Tamoxifen Treated
NRF1
66kDa
NRF2
66kDa
Complex IV – subunit 4
16kDa
Ponceau S staining
