# Supplementary material for: Vitamin D status modulates mitochondrial oxidative capacities in skeletal muscle: role in sarcopenia
Source: Commun Biol. 2022 Nov 24;5:1288. doi: 10.1038/s42003-022-04246-3 (PMC9700804; doi:10.1038/s42003-022-04246-3)
Supplement: Supplementary file 3 — Reporting Summary-New [file 42003_2022_4246_MOESM3_ESM.pdf]

nature portfolio

Corresponding author(s): Stephane Walrand

Last updated by author(s): Apr 14, 2022

Reporting Summary

Nature Portfolio wishes to improve the reproducibility of the work that we publish. This form provides structure for consistency and transparency in reporting. For further information on Nature Portfolio policies, see our Editorial Policies and the Editorial Policy Checklist.

Statistics

For all statistical analyses, confirm that the following items are present in the figure legend, table legend, main text, or Methods section.

n/a	Confirmed	
☐	☒	The exact sample size (*n*) for each experimental group/condition, given as a discrete number and unit of measurement
☐	☒	A statement on whether measurements were taken from distinct samples or whether the same sample was measured repeatedly
☐	☒	The statistical test(s) used AND whether they are one- or two-sided<br>*Only common tests should be described solely by name; describe more complex techniques in the Methods section.*
☒	☐	A description of all covariates tested
☒	☐	A description of any assumptions or corrections, such as tests of normality and adjustment for multiple comparisons
☐	☒	A full description of the statistical parameters including central tendency (e.g. means) or other basic estimates (e.g. regression coefficient) AND variation (e.g. standard deviation) or associated estimates of uncertainty (e.g. confidence intervals)
☐	☒	For null hypothesis testing, the test statistic (e.g. *F*, *t*, *r*) with confidence intervals, effect sizes, degrees of freedom and *P* value noted<br>*Give P values as exact values whenever suitable.*
☒	☐	For Bayesian analysis, information on the choice of priors and Markov chain Monte Carlo settings
☒	☐	For hierarchical and complex designs, identification of the appropriate level for tests and full reporting of outcomes
☒	☐	Estimates of effect sizes (e.g. Cohen's *d*, Pearson's *r*), indicating how they were calculated

Our web collection on statistics for biologists contains articles on many of the points above.

Software and code

Policy information about availability of computer code

Data collection	No software was used for data collection.
Data analysis	For microarray analysis, functional enrichment results were filtered and only the top 10 annotations for Gene Ontology BP, CC, KEGG and Reactome were kept for functional enrichment visualization using Cytoscape (v3.8) with EnrichmentMap and AutoAnnotate plugins.

For manuscripts utilizing custom algorithms or software that are central to the research but not yet described in published literature, software must be made available to editors and reviewers. We strongly encourage code deposition in a community repository (e.g. GitHub). See the Nature Portfolio guidelines for submitting code & software for further information.

Data

Policy information about availability of data

All manuscripts must include a data availability statement. This statement should provide the following information, where applicable:

- Accession codes, unique identifiers, or web links for publicly available datasets
- A description of any restrictions on data availability
- For clinical datasets or third party data, please ensure that the statement adheres to our policy

Microarray analysis data of rat plantaris muscle and C2C12 myotubes are available from the GEO database (GSE67274 & GSE64803, respectively).

Field-specific reporting

Please select the one below that is the best fit for your research. If you are not sure, read the appropriate sections before making your selection.

☒ Life sciences ☐ Behavioural & social sciences ☐ Ecological, evolutionary & environmental sciences

For a reference copy of the document with all sections, see nature.com/documents/nr-reporting-summary-flat.pdf

Life sciences study design

All studies must disclose on these points even when the disclosure is negative.

Sample size	For animal and cellular studies, sample sizes were determined based on published studies in the field or our previous experiences. For the human vitamin D supplementation study (interventional study), the sample size was calculated to detect the difference of appendicular skeletal muscle mass at 6 months (primary efficacy endpoint) between the two treatment arms with a two-sided type-I error of 0.05 and a power of 80%.
Data exclusions	No data were excluded from the analyses.
Replication	Each cell culture experiment was performed using two or three replicates. Cell culture data are combined from at least two independent experiments.
Randomization	For rat experiment, animals were randomly divided into two groups according to body weight, fat mass and lean mass. For mouse experiment 1 (Long-term vitamin D depletion), animals were randomly divided into two groups according to body weight and fasting glucose concentration. For mouse experiment 2 (Generation of human skeletal actin-MCM-VDRfl/fl transgenic mice), animals were randomly divided into two groups. The human interventional study, participants were randomized into two groups.
Blinding	For vitamin D supplementation, elderly subjects joined a randomized, controlled, double-blind intervention study. For both human studies, plasma and serum samples were coded. The analyses were therefore blinded. For animal and cell culture studies, analyses were not blinded.

Reporting for specific materials, systems and methods

We require information from authors about some types of materials, experimental systems and methods used in many studies. Here, indicate whether each material, system or method listed is relevant to your study. If you are not sure if a list item applies to your research, read the appropriate section before selecting a response.

Materials & experimental systems

n/a	Involved in the study	
☐	☒	Antibodies
☐	☒	Eukaryotic cell lines
☐	☐	Palaeontology and archaeology
☐	☒	Animals and other organisms
☐	☒	Human research participants
☐	☒	Clinical data
☐	☐	Dual use research of concern

Methods

n/a	Involved in the study	
☐	☐	ChIP-seq
☐	☐	Flow cytometry
☐	☐	MRI-based neuroimaging

Antibodies

Antibodies used	Primary antibodies used :<br>- Total OXPHOS Rodent WB Antibody Cocktail; ABCAM; Cat. Number : ab110413<br>- Complex IV subunit IV antibody; ThermoFisher Scientific; Cat. Number : A21348; Clone 20E8C12<br>- VDAC/Porin antibody; Biovision; Cat. Number : 3594<br>- NRF1 antibody; Genetex; Cat. Number : GTX103179<br>- NRF2 antibody; Genetex; Cat. Number : GTX103322<br>Horseradish peroxidase-conjugated secondary antibodies used :<br>- Polyclonal Swine Anti-Rabbit Immunoglobulins – HRP ; DAKO; Cat. Number : P039901<br>- Polyclonal Swine Anti-Mouse Immunoglobulins – HRP ; DAKO; Cat. Number : P044701
Validation	All primary antibodies we used were validated by the manufacturers for western-blot application. All antibodies we used were directed against mitochondrial proteins. We validated each of them with the Rat Heart mitochondria Western Blot Control provided with Total OXPHOS Rodent WB Antibody Cocktail (ABCAM; Cat. Number : ab110413).

Eukaryotic cell lines

Policy information about cell lines

Cell line source(s)	- C2C12 myoblasts; ATCC, Cat. Number : CRL-1772<br>- Cryopreserved Human Skeletal Myoblasts; Zenbio, Inc; Cat. Number : 088SKB-F; Lot SLSK002
Authentication	The cell lines we used were not authenticated since they were purchased from commercial source and were validated before shipping. We systematically assessed their growth and their morphology under microscope observation.
Mycoplasma contamination	C2C12 cell line was tested negative for mycoplasma contamination.<br>Human skeletal myoblasts from ZenBio were not tested for mycoplasma contamination.
Commonly misidentified lines (See ICLAC register)	No cell lines used in this study are listed in ICLAC v11.

Palaeontology and Archaeology

Specimen provenance	*Provide provenance information for specimens and describe permits that were obtained for the work (including the name of the issuing authority, the date of issue, and any identifying information). Permits should encompass collection and, where applicable, export.*
Specimen deposition	*Indicate where the specimens have been deposited to permit free access by other researchers.*
Dating methods	*If new dates are provided, describe how they were obtained (e.g. collection, storage, sample pretreatment and measurement), where they were obtained (i.e. lab name), the calibration program and the protocol for quality assurance OR state that no new dates are provided.*

☐ Tick this box to confirm that the raw and calibrated dates are available in the paper or in Supplementary Information.

Ethics oversight	*Identify the organization(s) that approved or provided guidance on the study protocol, OR state that no ethical approval or guidance was required and explain why not.*

Note that full information on the approval of the study protocol must also be provided in the manuscript.

Animals and other organisms

Policy information about studies involving animals; ARRIVE guidelines recommended for reporting animal research

Laboratory animals	Rat experiment (Long-term vitamin D depletion) : Male rattus norvegicus strain Wistar rats were 15-month-old at the beginning of the experiment.<br>Mouse experiment 1 (Long-term vitamin D depletion) : male C57BL/6j RJ mice were 5-month-old at the beginning of the experiment.<br>Mouse experiment 2 (Generation of human skeletal actin-MCM-VDRfl/fl transgenic mice) : Male and female human skeletal actin-MCM-VDRfl/fl transgenic mice were between 34 and 40 week-old at the beginning of tamoxifen or corn oil treatment.
Wild animals	The study did not involve wild animals.
Field-collected samples	The study did not involve samples collected from the field.
Ethics oversight	Rat experiment and Mouse experiment 2 : All animal procedures were approved by the institution's animal welfare committee (Comité d'Ethique en Matière d'Expérimentation Animale Auvergne: C2EA-02).<br>Mouse experiment 1: Procedures were conducted according to the institutional and national guidelines for the care and use of animal and were reviewed and approved by the Local Committee for Care and Use of Laboratory Animals at Wageningen University (code number: drs-2010123).

Note that full information on the approval of the study protocol must also be provided in the manuscript.

Human research participants

Policy information about studies involving human research participants

Population characteristics	Human trial 1 - Impact of vitamin D status (observational study): Healthy male subjects aged between 20 and 79 years and with a Body Mass Index between 21.4 and 34.2 kg/m2 were included in the study. Each subject had normal blood biochemical profile and was sedentary. Participants were not taking any medications liable to affect the parameters under study (i.e. corticosteroids, b-adrenergic blockers, lipid-lowering agents and anticoagulants).<br>Human trial 2 - vitamin D supplementation (interventional study): Elderly subjects (median age : 73.31±2.05 years) having vitamin deficiency (25(OH)D < 20 ng/ml as per Institute of Medicine (IOM) recommendations) and being presarcopenic (when skeletal muscle mass/height2 = 7.26 kg/m2 for males and 5.45 kg/m2 for females) were asked to join a randomized, controlled, double blind study. Exclusion criteria were patients with a history of type-2-diabetes, congestive heart failure, liver failure, renal failure, cancer, or taking oral hypoglycemic drugs, statin therapy, or patients having metabolic bone disease.
Recruitment	Human trial 1 - Impact of vitamin D status (observational study): Participants were recruited by the study team of the Unit of

Recruitment	Human Nutrition (UNH), University of Clermont Auvergne, Clermont-Ferrand, France, through advertisements in local newspapers, on the radio or television, with flyers at public places, general practioners and health care professionals, and from existing databases.<br>Human trial 2 - vitamin D supplementation (interventional study: The recruitment was performed in the outpatient clinics of Saint Charles hospital (Beirut, Lebanon).
Ethics oversight	Human trial 1 - Impact of vitamin D status (observational study): The study protocol was approved by the local Ethical Review Board of the General Clinical Research Center (GCRC) at Clermont-Ferrand, France, in accordance with the Declaration of Helsinki.<br>Human trial 2 - vitamin D supplementation (interventional study): The study protocol was approved by the Ethics Committee of the institutional review board at Saint Charles Hospital, Beirut, Lebanon, in accordance with the Declaration of Helsinki.

Note that full information on the approval of the study protocol must also be provided in the manuscript.

Clinical data

Policy information about clinical studies

All manuscripts should comply with the ICMJE guidelines for publication of clinical research and a completed CONSORT checklist must be included with all submissions.

Clinical trial registration	Human trial 1 - Impact of vitamin D status (observational study): ClinicalTrials.gov Identifier: NCT01066091<br><br>Human trial 2 - vitamin D supplementation (interventional study): ClinicalTrials.gov Identifier: NCT02942732
Study protocol	Human trial 1 - Impact of vitamin D status (observational study): Further details are provided in ClinicalTrials.gov Identifier: NCT01066091.<br><br>Human trial 2 - vitamin D supplementation (interventional study): Further details are provided in El Hajj et al. Effect of vitamin D treatment on glucose homeostasis and metabolism in Lebanese older adults: A randomized controlled trial. The Journal of Nutrition, Health & Aging. 2018;22(9):1128-1132; and in El Hajj et al. Vitamin D supplementation and muscle strength in pre-sarcopenic elderly Lebanese people: a randomized controlled trial. Archives of Osteoporosis. 2018;14(1):4.
Data collection	Human trial 1 - Impact of vitamin D status (observational study): Recruitment took place between March 2010 and January 2011 at the Center for Research in Human Nutrition Auvergne, Clermont-Ferrand, France.<br><br>Human trial 2 - vitamin D supplementation (interventional study): Recruitment took place between May 2015 and September 2015 at at the endocrinology and orthopedics outpatient clinics of Saint Charles Hospital (Fiyadiyeh-Lebanon) .
Outcomes	Human trial 1 - Impact of vitamin D status (observational study): Primary outcome measures:<br>Inflammatory profile by interleukin-6 (IL6) concentration in plasma. [Time Frame: six times per day (kinetic: T0 to T480) on three interventional days]<br><br>Human trial 2 - vitamin D supplementation (interventional study): Primary outcome measures:<br>1-Fasting blood glucose (mmol/l) [Time Frame: Baseline and 6 months] Change in Fasting blood glucose in mmol/l<br>2-HbA1C (%) [Time Frame: Baseline and 6 months] Change in HbA1C (%)<br>3-HOMA-IR [Time Frame: Baseline and 6 months] Change in HOMA-IR<br>4-Fasting Insulin (mIU/L) [Time Frame: Baseline and 6 months] Change in Fasting Insulin (mIU/L)

Dual use research of concern

Policy information about dual use research of concern

Hazards

Could the accidental, deliberate or reckless misuse of agents or technologies generated in the work, or the application of information presented in the manuscript, pose a threat to:

No	Yes	
☒	☐	Public health
☒	☐	National security
☒	☐	Crops and/or livestock
☒	☐	Ecosystems
☒	☐	Any other significant area

Experiments of concern

Does the work involve any of these experiments of concern:

No	Yes	
☒	☐	Demonstrate how to render a vaccine ineffective
☒	☐	Confer resistance to therapeutically useful antibiotics or antiviral agents
☒	☐	Enhance the virulence of a pathogen or render a nonpathogen virulent
☒	☐	Increase transmissibility of a pathogen
☒	☐	Alter the host range of a pathogen
☒	☐	Enable evasion of diagnostic/detection modalities
☒	☐	Enable the weaponization of a biological agent or toxin
☒	☐	Any other potentially harmful combination of experiments and agents

ChIP-seq

Data deposition

☐ Confirm that both raw and final processed data have been deposited in a public database such as GEO.

☐ Confirm that you have deposited or provided access to graph files (e.g. BED files) for the called peaks.

Data access links<br>*May remain private before publication.*	*For "Initial submission" or "Revised version" documents, provide reviewer access links. For your "Final submission" document, provide a link to the deposited data.*
Files in database submission	*Provide a list of all files available in the database submission.*
Genome browser session<br>(e.g. UCSC)	*Provide a link to an anonymized genome browser session for "Initial submission" and "Revised version" documents only, to enable peer review. Write "no longer applicable" for "Final submission" documents.*

Methodology

Replicates	*Describe the experimental replicates, specifying number, type and replicate agreement.*
Sequencing depth	*Describe the sequencing depth for each experiment, providing the total number of reads, uniquely mapped reads, length of reads and whether they were paired- or single-end.*
Antibodies	*Describe the antibodies used for the ChIP-seq experiments; as applicable, provide supplier name, catalog number, clone name, and lot number.*
Peak calling parameters	*Specify the command line program and parameters used for read mapping and peak calling, including the ChIP, control and index files used.*
Data quality	*Describe the methods used to ensure data quality in full detail, including how many peaks are at FDR 5% and above 5-fold enrichment.*
Software	*Describe the software used to collect and analyze the ChIP-seq data. For custom code that has been deposited into a community repository, provide accession details.*

Flow Cytometry

Plots

Confirm that:

☐ The axis labels state the marker and fluorochrome used (e.g. CD4-FITC).

☐ The axis scales are clearly visible. Include numbers along axes only for bottom left plot of group (a 'group' is an analysis of identical markers).

☐ All plots are contour plots with outliers or pseudocolor plots.

☐ A numerical value for number of cells or percentage (with statistics) is provided.

Methodology

Sample preparation	*Describe the sample preparation, detailing the biological source of the cells and any tissue processing steps used.*
Instrument	*Identify the instrument used for data collection, specifying make and model number.*

Software | *Describe the software used to collect and analyze the flow cytometry data. For custom code that has been deposited into a community repository, provide accession details.*
Cell population abundance | *Describe the abundance of the relevant cell populations within post-sort fractions, providing details on the purity of the samples and how it was determined.*
Gating strategy | *Describe the gating strategy used for all relevant experiments, specifying the preliminary FSC/SSC gates of the starting cell population, indicating where boundaries between "positive" and "negative" staining cell populations are defined.*

☐ Tick this box to confirm that a figure exemplifying the gating strategy is provided in the Supplementary Information.

Magnetic resonance imaging

Experimental design

Design type | *Indicate task or resting state; event-related or block design.*
Design specifications | *Specify the number of blocks, trials or experimental units per session and/or subject, and specify the length of each trial or block (if trials are blocked) and interval between trials.*
Behavioral performance measures | *State number and/or type of variables recorded (e.g. correct button press, response time) and what statistics were used to establish that the subjects were performing the task as expected (e.g. mean, range, and/or standard deviation across subjects).*

Acquisition

Imaging type(s) | *Specify: functional, structural, diffusion, perfusion.*
Field strength | *Specify in Tesla*
Sequence & imaging parameters | *Specify the pulse sequence type (gradient echo, spin echo, etc.), imaging type (EPI, spiral, etc.), field of view, matrix size, slice thickness, orientation and TE/TR/flip angle.*
Area of acquisition | *State whether a whole brain scan was used OR define the area of acquisition, describing how the region was determined.*
Diffusion MRI ☐ Used ☐ Not used

Preprocessing

Preprocessing software | *Provide detail on software version and revision number and on specific parameters (model/functions, brain extraction, segmentation, smoothing kernel size, etc.).*
Normalization | *If data were normalized/standardized, describe the approach(es): specify linear or non-linear and define image types used for transformation OR indicate that data were not normalized and explain rationale for lack of normalization.*
Normalization template | *Describe the template used for normalization/transformation, specifying subject space or group standardized space (e.g. original Talairach, MNI305, ICBM152) OR indicate that the data were not normalized.*
Noise and artifact removal | *Describe your procedure(s) for artifact and structured noise removal, specifying motion parameters, tissue signals and physiological signals (heart rate, respiration).*
Volume censoring | *Define your software and/or method and criteria for volume censoring, and state the extent of such censoring.*

Statistical modeling & inference

Model type and settings | *Specify type (mass univariate, multivariate, RSA, predictive, etc.) and describe essential details of the model at the first and second levels (e.g. fixed, random or mixed effects; drift or auto-correlation).*
Effect(s) tested | *Define precise effect in terms of the task or stimulus conditions instead of psychological concepts and indicate whether ANOVA or factorial designs were used.*
Specify type of analysis: ☐ Whole brain ☐ ROI-based ☐ Both
Statistic type for inference (See Eklund et al. 2016) | *Specify voxel-wise or cluster-wise and report all relevant parameters for cluster-wise methods.*
Correction | *Describe the type of correction and how it is obtained for multiple comparisons (e.g. FWE, FDR, permutation or Monte Carlo).*

Models & analysis

n/a	Involved in the study
☐	☐ Functional and/or effective connectivity
☐	☐ Graph analysis
☐	☐ Multivariate modeling or predictive analysis

Functional and/or effective connectivity	*Report the measures of dependence used and the model details (e.g. Pearson correlation, partial correlation, mutual information).*
Graph analysis	*Report the dependent variable and connectivity measure, specifying weighted graph or binarized graph, subject- or group-level, and the global and/or node summaries used (e.g. clustering coefficient, efficiency, etc.).*
Multivariate modeling and predictive analysis	*Specify independent variables, features extraction and dimension reduction, model, training and evaluation metrics.*
